# Joint Beamforming and Phase Shifts Design for RIS-Aided Multi-User Full-Duplex Systems in Smart Cities

**DOI:** 10.3390/s24010121

**Published:** 2023-12-25

**Authors:** Kunbei Pan, Bin Zhou, Wei Zhang, Cheng Ju

**Affiliations:** 1 Shanghai Institute of Microsystem and Information Technology, Chinese Academy of Sciences, Shanghai 200050, China; pankunbei@mail.sim.ac.cn (K.P.); wzhang@mail.sim.ac.cn (W.Z.); cheng.ju@mail.sim.ac.cn (C.J.); 2 University of Chinese Academy of Sciences, Beijing 100049, China

**Keywords:** full-duplex, reconfigurable intelligent surface, spectral efficiency, beamforming, phase shifts, deep reinforcement learning, urban outdoor environment

## Abstract

Full-duplex (FD) and reconfigurable intelligent surface (RIS) are potential technologies for achieving wireless communication effectively. Therefore, in theory, the RIS-aided FD system is supposed to enhance spectral efficiency significantly for the ubiquitous Internet of Things devices in smart cities. However, this technology additionally induces the loop-interference (LI) of RIS on the residual self-interference (SI) of the FD base station, especially in complicated urban outdoor environments, which will somewhat counterbalance the performance benefit. Inspired by this, we first establish an objective and constraints considering the residual SI and LI in two typical urban outdoor scenarios. Then, we decompose the original problem into two subproblems according to the variable types and jointly design the beamforming matrices and phase shifts vector methods. Specifically, we propose a successive convex approximation algorithm and a soft actor–critic deep reinforcement learning-related scheme to solve the subproblems alternately. To prove the effectiveness of our proposal, we introduce benchmarks of RIS phase shifts design for comparison. The simulation results show that the performance of the low-complexity proposed algorithm is only slightly lower than the exhaustive search method and outperforms the fixed-point iteration scheme. Moreover, the proposal in scenario two is more outstanding, demonstrating the application predominance in urban outdoor environments.

## 1. Introduction

With the recent progress in requirement definitions and developing potential technologies of the sixth generation (6G) wireless communication, the capacity of the 6G network will increase by nearly 1000 times compared with the fifth generation (5G) to support the ubiquitous Internet of Things (IoT) devices [1,2,3]. Full-duplex (FD) technology (i.e., in-band co-time co-frequency) can double the spectral efficiency (SE) at most compared with the traditional time division duplex (TDD) or frequency division duplex (FDD). Thus, it is regarded as one of the Beyond 5G/6G candidate technologies [1,4,5,6]. On the other hand, the reconfigurable intelligent surface (RIS) has reflection elements with programmable super-atomic structure and ultra-low power consumption, which can manipulate the signals’ reflection and scattering scenes to improve coverage and quality of service (QoS). Moreover, it can reduce energy consumption compared with conventional relays [7]. In view of the revolutionary technology that endows network entities with reconfigurable properties, RIS has become a promising technology for 6G networks [8,9] and could be extensively applied in the coverage tribulations circumstances [10,11]. Overall, the united technology of FD and RIS can theoretically obtain two-fold performance gains, including time-frequency domain multiplexing and signal enhancement. So, RIS-aided FD technology is expected to be applied in a wide range of IoT devices in smart cities that require superior performances in terms of high data transmission rate and extensive coverage [3,12].

It is known that FD technology is limited by strong self-interference (SI). Although the existing SI cancellation technology (i.e., active cancellation and passive cancellation) [5] can eliminate the SI to approximate the noise floor [13], residual SI still exists due to hardware impairment [14]. With the RIS introduced, the residual SI can be mitigated to a degree. However, the FD system additionally affiliates with the loop-interference (LI) caused by the transmit signal rebounded via RIS unexpectedly. Particularly when IoT devices are located in complicated urban outdoor environments, including severe attenuation, reflections, and blockages [15], these two types of interference tend to occur frequently, which will not give full play to the potential performance of the RIS-aided FD systems.

Scholars often model an SE or energy efficiency (EE) optimization to overcome interference-related issues. To tackle the residual SI in the FD system, the scholars of [16] constructed a maximum SE problem concerning sub-carrier and power allocation. They transformed the non-convex problem into a convex problem through first-order Taylor approximation. The authors of [17,18] mainly studied the allocation of FD antennas and the design of beamforming strategy to construct sum rate maximization problems. Specifically, in [17], the maximum ratio transmission and genetic algorithms were used to solve the beamforming and antenna selection concerned subproblems, respectively. The authors in [18] devised a block coordinate descent method to solve the multi-variable optimization problem alternately. Refs. [16,17,18] proved that the optimized FD system with residual SI was still superior to the half-duplex (HD) system, such as TDD, in improving the performance of SE. Meanwhile, RIS is a disruptive technology that is greatly concerned by many scholars. Therefore, the scholars of [19] introduced RIS to formulate a target optimization problem with respect to channel cascade. They obtained the global EE optimum by alternating optimization of fixed variables. Other scholars applied RIS to FD/HD relay scenarios [20]. They established a problem of minimizing the required power of the base station (BS) and relay to enhance EE, where the QoS constraints were also covered. To solve the two scenarios involved problems, they proposed the semi-definite programming and the maximum weakest hop-signal–noise-ratio methods. Simulation results showed that RIS joined with the FD relay was superior to other cases. Based on the previous inferences, the authors of [21,22,23,24,25,26] considered the union of RIS and FD technology and demonstrated the performance gains. To name a few, the authors of [21] proposed an SI mitigation method in RIS-assisted FD system, thus mitigating the strong SI to feed in the analog-to-digital converters of finite bit resolutions. The authors of [22] formulated the mathematical expressions of outage probability and ergodic capacity. They concluded that RIS could indirectly diminish the adverse consequence of the residual SI and ameliorate the performance in the FD system. Other scholars designed the minimized transmit power objective with active and passive beamforming to strengthen EE by suppressing interference [23]. The work of [24] discussed the influence of different numbers of reflection elements and receiving antennas on FD system performance.

Deep reinforcement learning (DRL) can allow a wireless communication system agent to seek the optimal policy by observing the reward without a priori knowledge. Accordingly, the mathematically intractable problems could be settled by the agent interacting with the environment [27,28,29]. The authors established the objective by considering SE and energy harvest in the FD system [30]. Then, they devised a hybrid deep deterministic policy gradient and deep double Q-learning network approach to train the networks regarding different variables, respectively. Based on DRL, the authors of [31] proposed a maximizing entropy scheme to solve active and passive beamforming. In [32], neural epsilon-greedy, deep Q-learning network, upper confidence bound, and other DRL approaches were considered. They took the sum rate of the RIS system as the objective and proved that the trained artificial neural network (NN) could improve performance compared with some traditional non-convex algorithms on certain occasions. The authors of [25] employed a DRL method to work out the single and distributed RIS-related issues. They demonstrated that DRL could reduce the optimum performance loss without pre-relaxation.

Although several previous studies have focused on the performance enhancement of FD RIS-aided systems, they did not discuss the influence of LI with different user wireless environments and RIS locations. More importantly, to the best of our knowledge, there are no two-step algorithms combined with a closed-form solution and a learning method in multi-user RIS-aided FD systems in the previous works. Furthermore, the discrete phase shifts model of RIS is consistent with the RIS physical realization, which is more practical than the continuous phase shifts model. Motivated by this, we have studied the RIS-aided FD system with discrete phase shifts, considering the effects of residual SI, LI, and RIS location in different urban outdoor scenarios. Then, we devise a two-step solution to the formulated non-convex problem. The main contributions are as follows:

- We introduce a discrete phase shifts RIS model in the blockage of the line-of-sight (LoS) outdoor environment of smart cities. The objective and constraints concerning the residual SI and LI are formulated according to two typical scenarios. Under the two scenarios, we can focus on the influence of the primary interference. Next, a two-step algorithm based on the variable types is proposed, and we can further emphasize the proposal advantage through the scenario handoff.
- Specifically, the original optimization problem is decomposed into two subproblems in light of the type of variables. We attempt to optimize the transmitting and receive beamforming matrices through fixed phase shifts in subproblem one. Due to the non-convexity of this problem, we design a novel successive convex approximation (SCA) method to obtain the approximate convex lower bound of the objective function and constraints. Therefore, the original non-convex problem is transformed into a convex one that can be directly solved.
- Then, we tackle subproblem two to optimize the phase shifts vector via given beamforming matrices. In view of the non-convex optimization for the discrete variable to be solved, we develop a discrete soft actor–critic (SAC) algorithm based on DRL. We seek to maximize the reward to obtain the optimal sum SE by defining the corresponding action, state, and reward. Remarkably, our devised DRL-based method only involves discrete phase shifts, dramatically reducing the dimensions of the action space. Additionally, the state of the environment is a vector consisting of signal to interference-plus-noise ratio (SINR) of each IoT device, which can be efficiently applied to the multi-user case.
- Finally, after iteratively optimizing the beamforming matrices and phase shifts vector, we evaluate the performance of the proposed algorithm from various perspectives and draw relevant conclusions. To be specific, the extensive simulation results show that the low-complexity proposal performance is second only to the exhaustive search method and outweighs the fixed-point iteration baseline. Particularly, the proposed algorithm performs outstandingly in scenario two, demonstrating the superiority of our proposal to mitigate interference in the complicated urban outdoor environment.

The rest of this paper is organized as follows. The system model and problem formulation are described in Section 2. Section 3 presents our proposal that concerns a joint beamforming and phase shifts design. The computational complexity is also provided. Section 4 discusses numerical results to evaluate the performance of the proposed algorithm. A conclusion and future issues are given in Section 5.

Notations: For a general matrix **A**, **A***^H^*, and ‖A‖ denote the Hermitian and Frobenius norm of **A**. The subscript/superscript t, r, d, and u indicate transmitting, receiving, downlink (DL), and uplink (UL) related. E[⋅] represents the expectation. We signify the conjugate and real part of a complex number by ${\left(\cdot \right)}^{*}$ and Re(⋅), respectively. For a general vector **x**, diag{**x**} denotes a diagonal matrix with diagonal elements **x**. The modulus of **x** is denoted by |x|. ${\mathbb{C}}^{a\times b}$ represents the space of a×b complex number matrix. **1** and **0** denote a vector or matrix where all elements are one and zero. An identity matrix is represented by the letter **I**. CN denotes a complex Gaussian distribution. Other calligraphy upper-case letters, such as J and K, stand for the sets. The letter of superscript apostrophes, such as $k^{\prime }$, indicates the element of the supplementary set of {k∈K, $k\neq k^{\prime }$}. The partial derivative of f(x) to *x* is signified by $\nabla_{x}f(x)$.

The acronyms used in this paper are given in Table 1.

## 2. System Model and Problem Formulation

**Figure 1:**
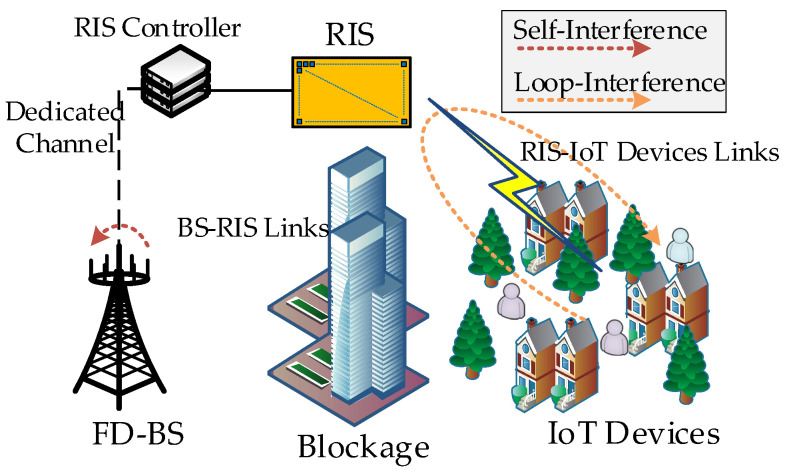
A multi-user RIS-aided full-duplex system in urban outdoor environment.

In this section, we first describe a multi-user RIS-aided FD system, as presented in Figure 1, exploiting RIS to promote FD performance in the blockage of LoS urban outdoor scenario. Then, by describing the transmission model of two typical urban outdoor scenarios, we focus on the problem of maximizing the sum SE in the simultaneous DL and UL data transmission. The problem formulation is characterized as a joint design of transmitting and receive beamforming matrices and phase shifts vector.

### 2.1. System Overview

Figure 1 describes a multi-user RIS-aided FD system, which composes one BS equipped with $N_{t}$ transmitting and $N_{r}$ receiving antennas, (*J* + *K*) single-antenna IoT devices, and one RIS. BS works in FD mode, while the IoT devices, in terms of service type, are divided into *J* UL IoT devices and *K* DL IoT devices, operating in HD mode. The sets of UL and DL IoT devices are represented by J={1,2,…,J} and K= {1,2,…,K}, where we let IoT*_j_* (∀j∈J) and IoT*_k_* (∀k∈K) denote the *j*-th UL IoT device and *k*-th DL IoT device for simplification, respectively. Assuming that the BS and all IoT devices are entirely blocked by obstacles such as large buildings, the direct link between them can be ignored due to unfavorable transmission conditions [33]. We suppose that a RIS with *L* reflection elements is deployed beyond the obstacles. With the help of the RIS, we can maintain the direct links of BS-RIS and RIS-IoT devices, thus controlling the respective transmission wave characteristics, such as scattering, reflection, and refraction. In this way, RIS can reconstruct and enhance the desired signal for BS and devices [34]. Meanwhile, the controller connects with the RIS to programmatically operate each reflection element and communicates with BS through a dedicated channel.

As shown in Figure 1, the BS-RIS DL channel matrix, RIS-BS UL channel matrix, RIS-IoT*_k_* DL channel vector, and IoT*_j_*-RIS UL channel vector are denoted as $H_{d}\in {\mathbb{C}}^{N_{t}\times L}$, $H_{u}\in {\mathbb{C}}^{N_{r}\times L}$, $h_{k}^{d}\in {\mathbb{C}}^{L\times 1}$ and $h_{j}^{u}\in {\mathbb{C}}^{L\times 1}$, respectively. The BS residual SI matrix is represented as $H_{\mathrm{SI}}\in {\mathbb{C}}^{N_{t}\times N_{r}}$. We assume that the above channel state information (CSI) of all channels involved is known, which allows us to investigate the upper bounds for the performance [35,36]. The *l*-th reflection element’s coefficient is regarded as $\beta_{l}e^{j\phi_{l}}$, where *l* belongs to the set ℒ={1,2,…,L} of reflection elements. $\beta_{l}$ and $\phi_{l}$ are the related amplitude and phase. Considering that the reflection element is a passive device without an external power amplifier, we usually let $\beta_{l}=1$ [37]. Thus, the diagonal matrix of coefficients is denoted as $\Phi =\mathrm{diag}\{e^{j\phi_{1}},e^{j\phi_{2}},\ldots ,e^{j\phi_{L}}\}\in {\mathbb{C}}^{L\times L}$. For the convenience of matrix operation in this paper, we fetch the diagonal entries of Φ and reshape a new phase shifts vector $\phi =[e^{j\phi_{1}},e^{j\phi_{2}},\ldots ,$ $e^{j\phi_{L}}]\in {\mathbb{C}}^{1\times L}$. Note that, the phase shifts are discrete values limited by the diode mechanism in engineering practice, such as {0, $2\pi /2^{b},\;$$2\cdot 2\pi /2^{b},$ $\ldots ,(2^{b}-1)\cdot 2\pi /2^{b}\}$, where the phase shift resolution *b* determines the phase shift accuracy.

### 2.2. Transmission Model

In this subsection, we focus on the DL/UL data transmission process.

#### 2.2.1. DL Transmission Model

Since we introduce LI of RIS in this paper (see Figure 1 on the IoT devices side), the received signal at IoT*_k_* is expressed as

(1) $$y_{k}^{d}=\phi H_{\mathrm{BR},k}w_{k}x_{k}^{d}+\sum_{k^{\prime }\in K,k^{\prime }\neq k}\phi H_{\mathrm{BR},k}w_{k^{\prime }}x_{k^{\prime }}^{d}+\sum_{j\in J}g_{k,j}\sqrt{p}x_{j}^{u}+\sum_{j\in J}\phi H_{\mathrm{LI},j,k}\sqrt{p}x_{j}^{u}+n_{k}^{d},$$

where

(2a) $$H_{\mathrm{BR},k}=\mathrm{diag}(h_{k}^{d})H_{d}^{H}\in {\mathbb{C}}^{L\times N_{t}},$$

(2b) $$H_{\mathrm{LI},j,k}=\mathrm{diag}(h_{k}^{d})h_{j}^{u}\in {\mathbb{C}}^{L\times 1}.$$

$H_{\mathrm{BR},k}$ and $H_{\mathrm{LI},j,k}$ represent the BS-RIS-IoT*_k_* and IoT*_j_*-RIS-IoT*_k_* cascade channels. $w_{k}\in {\mathbb{C}}^{N_{t}\times 1}$ is the transmitting beamforming vector for IoT*_k_*, and the modulus of the vector determines the allocated power level to IoT*_k_*. $x_{k}^{d}$ and $x_{j}^{u}$ indicate the receiving and transmitting symbols of IoT*_k_* and IoT*_j_*, respectively, which satisfy $E[x_{k}^{d}{\left(x_{k}^{d}\right)}^{*}]=1$ and $E[x_{j}^{u}{\left(x_{j}^{u}\right)}^{*}]=1$. *p* is the transmit power of UL IoT devices. We suppose each device adopts the full power *p* for transmission. $g_{k,j}$ is the channel gain between IoT*_k_* and IoT*_j_*. $n_{k}^{d}$ stands for the additive white gaussian noise (AWGN) at IoT*_k_*, which follows $n_{k}^{d}∼$$CN\left(0,\sigma_{d,k}^{2}\right)$. $\sigma_{d,k}^{2}$ is the variance of noise power.

In (1), the first term represents the desired received signal at IoT*_k_*. The second and third terms mean the regular DL and UL co-channel multi-user interference without the unexpected rebound signal. Unlike the common interference, the fourth term denotes LI caused by UL transmitted signals rebounding on the IoT devices side.

We observe that (1) contains several types of interference. The mixture of different kinds of interference is not conducive to studying the respective influencing factors of performance. Thus, the DL transmission model can be approximated in terms of two typical scenarios, according to the actual geographical location of IoT devices in a complicated urban outdoor environment, as follows.

- Scenario one: The IoT devices are relatively open to each other in a local region like a town square, where no barriers are between them.
- Scenario two: The IoT devices are located in a residential area and separated by small-sized obstacles, such as low residences and trees (Though the real situation in smart cities may be a hybrid of scenarios one and two, the research through two typical cases under extreme conditions can well extend to the general situation).

To be specific, in scenario one, even the RIS located at the IoT devices side, the direct IoT*_j_*-IoT*_k_* path loss is smaller than the IoT*_j_*-RIS-IoT*_k_* path loss of loopback due to the double fading effect. The influence of the third term of (1) is greater than the fourth term, which becomes the dominating factor of performance deterioration. Considering the subordinate influence of LI among interference and the insignificant performance improvement by deliberately optimizing RIS for restraining LI in scenario one, we can weaken the LI effect and approximate the reconfiguration of LI by RIS as a constant term for ease of simplicity. Thus the LI is approximated as an equivalent AWGN, whose intensity depends on the average distance between RIS and IoT devices. On the contrary, for scenario two, the channel gains between IoT devices are relatively small because of the scattered small-sized obstructions, so the direct link interference of UL to DL IoT devices (simplified as UL to DL interference (UDI) below) can be seen as equivalent AWGN in comparison to LI. On this occasion, the effect of LI should be elaborately studied. Overall, scenarios one and two mainly focus on UDI and LI, respectively, which lets us do careful research about the influence of different major interference and not overlook either interference.

In the light of scenarios one and two, (1) can be rewritten as

(3a) $$y_{k,1}^{d}=\phi_{\;1}H_{\mathrm{BR},k}w_{k,1}x_{k}^{d}+\sum_{k^{\prime }\in K,k^{\prime }\neq k}\phi_{\;1}H_{\mathrm{BR},k}w_{k^{\prime },1}x_{k^{\prime }}^{d}+\sum_{j\in J}g_{k,j}\sqrt{p}x_{j}^{u}+{\hat{n}}_{k,1}^{d},$$

(3b) $$y_{k,2}^{d}=\phi_{\;2}H_{\mathrm{BR},k}w_{k,2}x_{k}^{d}+\sum_{k^{\prime }\in K,k^{\prime }\neq k}\phi_{\;2}H_{\mathrm{BR},k}w_{k^{\prime },2}x_{k^{\prime }}^{d}+\sum_{j\in J}\phi_{\;2}H_{\mathrm{LI},j,k}\sqrt{p}x_{j}^{u}+{\hat{n}}_{k,2}^{d},$$

where $y_{k,i}^{d}$, $w_{k,i}$, and $\phi_{\;i}$ represent the received signal, the transmitting beamforming vector, and the phase shifts vector in scenario *i*(i∈{1,2}). ${\hat{n}}_{k,i}^{d}$ denotes the aggregated AWGN of IoT*_k_*, detailed as

(4a) $${\hat{n}}_{k,1}^{d}=n_{k}^{d}+n_{\mathrm{LI},k},$$

(4b) $${\hat{n}}_{k,2}^{d}=n_{k}^{d}+n_{\mathrm{UDI},k},$$

where $n_{\mathrm{LI},k}$ and $n_{\mathrm{UDI},k}$ indicate the equivalent AWGN of LI and UDI in scenarios one and two, respectively.

#### 2.2.2. UL Transmission Model

As the SI at BS cannot be eliminated absolutely, the received signal of IoT*_j_* at BS is expressed as

(5) $$y_{j}^{u}=H_{j,\mathrm{RB}}\phi^{H}\sqrt{p}x_{j}^{u}+\sum_{j^{\prime }\in J,j^{\prime }\neq j}H_{j^{\prime },\mathrm{RB}}\phi^{H}\sqrt{p}x_{j^{\prime }}^{u}+\sum_{k\in K}H_{u}\Phi H_{d}^{H}w_{k}x_{k}^{d}+\sum_{k\in K}H_{\mathrm{SI}}^{H}w_{k}x_{k}^{d}+n_{j}^{u},$$

where $H_{j,\mathrm{RB}}=H_{u}\mathrm{diag}(h_{j}^{u})\in {\mathbb{C}}^{N_{r}\times L}$ denotes the IoT*_j_*-RIS-BS cascade channel matrix. $n_{j}^{u}$ and $H_{\mathrm{SI}}$ represent the AWGN of IoT*_j_* and the residual SI matrix at BS, respectively, which follow $n_{j}^{u}\in {\mathbb{C}}^{N_{r}\times 1}∼CN(0,\sigma_{u,k}^{2}1)$ and $H_{\mathrm{SI}}∼CN(\sigma_{\mathrm{SI}}{\left(a/(a+1)\right)}^{1/2}1,\sigma_{\mathrm{SI}}^{2}I/(a+1))$. *a* is the rician factor and $\sigma_{\mathrm{SI}}^{2}$ is the SI power elimination level [38].

Similar to (1), the first three terms of (5) indicate the desired signal, regular multi-user interference, and LI on the BS side, while the fourth term represents the residual SI at BS. It is worth noting that the second term-related interference produced at the BS side has the same form in each scenario. This varies from the DL received signal.

Due to the connection of the RIS controller and BS via the dedicated channel, BS can obtain the RIS reflection coefficients to effectively remove the LI on the BS side [26]. Thus, the third term of (5) can be ignored. For this reason, we also do not display LI on the BS side in Figure 1. So (5) can be simplified as

(6) $$y_{j}^{u}=H_{j,\mathrm{RB}}\phi^{H}\sqrt{p}x_{j}^{u}+\sum_{j^{\prime }\in J,j^{\prime }\neq j}H_{j^{\prime },\mathrm{RB}}\phi^{H}\sqrt{p}x_{j^{\prime }}^{u}+\sum_{k\in K}H_{\mathrm{SI}}^{H}w_{k}x_{k}^{d}+n_{j}^{u}.$$

Since the subscript *i,* standing for scenario one or two, only determines the last two terms of (3a) or (3b), respectively, we omit the subscript *i* in the other terms for formula conciseness in the following parts.

#### 2.2.3. Problem Formulation

Next, for the proposed RIS-aided FD system, we formulate the problem of SE maximation considering the joint optimization of transmitting and receive beamforming matrices and phase shifts vector under the two typical scenarios.

The DL SINR of IoT*_k_* is denoted as

(7) $$\gamma_{k}^{d}=\frac{{\left|\phi H_{\mathrm{BR},k}w_{k}\right|}^{2}}{\Psi_{1,k}^{d}+\Psi_{2,k,i}^{d}+\Psi_{3,k,i}^{d}},$$

where

(8a) $$\Psi_{1,k}^{d}=\sum_{k^{\prime }\in K,k^{\prime }\neq k}{\left|\phi H_{\mathrm{BR},k}w_{k^{\prime }}\right|}^{2},$$

(8b) $$\Psi_{2,k,i}^{d}=\{\begin{array}{l} p\sum_{j\in J}g_{k,j}^{2},\;i=1,\\ \sigma_{\mathrm{UDI},k}^{2},\;i=2, \end{array}$$

(8c) $$\Psi_{3,k,i}^{d}=\{\begin{array}{l} \sigma_{\mathrm{LI},k}^{2}+\sigma_{d,k}^{2},\;i=1,\\ p\sum_{j\in J}{\left|\phi H_{\mathrm{LI},j,k}\right|}^{2}+\sigma_{d,k}^{2},\;i=2. \end{array}$$

$\Psi_{1,k}^{d}$ represents the DL-to-DL IoT device interference power. $\Psi_{2,k,i}^{d}$ denotes the UDI power. Meanwhile, $\Psi_{3,k,i}^{d}$ indicates the LI and AWGN power aggregated together.

Similarly, the UL SINR of IoT*_j_* is represented as

(9) $$\gamma_{j}^{u}=\frac{p{\left|u_{j}^{H}H_{j,\mathrm{RB}}\phi^{H}\right|}^{2}}{\Psi_{1,j}^{u}+\Psi_{\mathrm{SI},j}+\sigma_{u,j}^{2}{\left|u_{j}\right|}^{2}},$$

where

(10a) $$\Psi_{1,j}^{u}=p\sum_{j^{\prime }\in J,j^{\prime }\neq j}{\left|u_{j}^{H}H_{j^{\prime },\mathrm{RB}}\phi^{H}\right|}^{2},$$

(10b) $$\Psi_{\mathrm{SI},j}=\sum_{k\in K}{\left|u_{j}^{H}H_{\mathrm{SI}}^{H}w_{k}\right|}^{2}$$

$\Psi_{1,j}^{u}$ and $\Psi_{\mathrm{SI},j}$ denote the UL to UL IoT device interference and residual SI power. $u_{j}\in {\mathbb{C}}^{N_{r}\times 1}$ is the receive beamforming vector for IoT*_j_*.

According to Shannon formula, the SE of IoT*_k_* and IoT*_j_* are recorded as

(11a) $$R_{k}^{d}\left(W,\phi \right)=\log\left(1+\gamma_{k}^{d}\right),$$

(11b) $$R_{j}^{u}\left(W,u_{j},\phi \right)=\log\left(1+\gamma_{j}^{u}\right),$$

where we signify transmitting beamforming matrix by $W=[w_{1},w_{2},\ldots ,w_{K}]\in {\mathbb{C}}^{N_{t}\times K}$.

Then, the sum SE is expressed as

(12) $$R\left(W,U,\phi \right)=\sum_{k\in K}R_{k}^{d}\left(W,\phi \right)+\sum_{j\in J}R_{j}^{u}\left(W,u_{j},\phi \right),$$

where $U=[u_{1},u_{2},\ldots ,u_{J}]\in {\mathbb{C}}^{N_{r}\times J}$ represents the receive beamforming matrix.

Mathematically, the SE maximization problem is formulated as

(13a) $$𝒫1:\underset{W,U,\phi }{\max}R\left(W,U,\phi \right)$$

(13b) s.t. i∈{1,2},

(13c) $$\sum_{k\in K}{\left|w_{k}\right|}^{2}\leq P,$$

(13d) |ϕ(l)|=1,1≤l≤L,

(13e) $$R_{k}^{d}\left(W,\phi \right)\geq R_{\mathrm{req}}^{d},\forall k\in K,$$

(13f) $$R_{j}^{u}\left(W,u_{j},\phi \right)\geq R_{\mathrm{req}}^{u},\forall j\in J,$$

where constraint (13b) determines the urban outdoor scenario in which the IoT devices are located. Constraint (13c) gives the BS power budget. (13d) represents the unit-modulus constraint for each reflection element of RIS. Constraints (13e) and (13f) ensure the QoS of IoT*_k_* and IoT*_j_*. It can be seen that the objective function (13a) involves a logarithmic function that includes fractions, and so do the constraints (13e) and (13f). Therefore, 𝒫1 with non-convex objective and constraints is intractable to optimize by conventional methods.

## 3. Solution to SE Maximization Problem in RIS-Aided FD System

Considering that the variables involved in 𝒫1 can be classified into continuity (such as **W**, **U** with continuous weights) and discreteness (such as ϕ with discrete phase shifts), it motivates us to adopt different schemes to solve problems with respect to different variable characteristics. Consequently, we decompose 𝒫1 into two subproblems to simplify and find the two-step solution through iteration until convergence. The rest of this section describes the optimization of subproblems one and two in developing beamforming and phase shifts, respectively.

### 3.1. Beamforming Design

Given the phase shifts vector $\phi^{*}$, the $\Psi_{3,k,i}^{d}$ in objective function is a constant, and unit-modulus constraint (13d) does not need to be considered. Thus, the design of beamforming matrices can be transformed into the following expression.

(14a) $$𝒫2:\underset{W,U}{\max}\sum_{k\in K}\log\left(1+\frac{{\left|\phi^{*}H_{\mathrm{BR},k}w_{k}\right|}^{2}}{\Psi_{1,k}^{d}+A_{k,i}}\right)+\sum_{j\in J}\log\left(1+\frac{p{\left|u_{j}^{H}H_{j,\mathrm{RB}}{\left(\phi^{*}\right)}^{H}\right|}^{2}}{\Psi_{1,j}^{u}+\Psi_{\mathrm{SI},j}+\sigma_{u,j}^{2}{\left|u_{j}\right|}^{2}}\right)$$

(14b) $$s.t.\;R_{k}^{d}\left(W\right)\geq R_{\mathrm{req}}^{d},\forall k\in K,$$

(14c) $$R_{j}^{u}\left(W,u_{j}\right)\geq R_{\mathrm{req}}^{u},\forall j\in J,$$

(13b), (13c), where

(15) $$A_{k,i}=\{\begin{array}{l} p\sum_{j\in J}g_{k,j}^{2}+\sigma_{\mathrm{LI},k}^{2}+\sigma_{d,k}^{2},\;i=1,\\ \sigma_{\mathrm{UDI},k}^{2}+p\sum_{j\in J}{\left|\phi^{*}H_{\mathrm{LI},k}\right|}^{2}+\sigma_{d,k}^{2},\;i=2. \end{array}$$

Obviously, the DL SE depends on the transmitting beamforming matrix. However, the UL SE is up to the receive and the residual SI-related transmitting beamforming, which shows the high coupling relationship between **W** and **U**. Therefore, 𝒫2 makes its solution challenging due to the non-convex objective function (14a) and constraints (14b), (14c).

In view of the two coupled variables, the core idea is to combine alternating optimization with SCA to transform the non-convexity into convexity. Motivated by [39], we can approximate the lower bound by inequality transformation to acquire the convexity as follows.

(16a) log(1+|x|2y)≥log(1+|x(n)|2y(n))−|x(n)|2y(n)+2Re{x(n)x}y(n)−|x(n)|2(|x|2+y)y(n)(y(n)+|x(n)|2),

(16b) xHYxy≥2Re{(x(n))HYx}y(n)−(x(n))HYx(n)y|y(n)|2,

where y>0, $y^{\left(n\right)}>0$.

First, we introduce an auxiliary variable set $\left\{\lambda_{k}\right\}$ to help find the lower bounds of the SINR of the DL IoT devices, which satisfies

(17a) $$\left|\phi^{*}H_{\mathrm{BR},k}w_{k}\right|\geq \left|\lambda_{k}\right|$$

(17b) $$\gamma_{k}^{d}\geq \frac{\lambda_{k}^{2}}{\Psi_{1,k}^{d}+A_{k,i}},$$

where $\lambda_{k}$ indicates the lower bound of the intended signal of IoT*_k_*.

Given the feasible point $w_{k}^{\left(n\right)}$ at the *n*-th iteration, we can acquire the boundary of $\lambda_{k}$ by (16b) and (17a).

(18) λk2≤(wk(n))HHBR,kH(ϕ∗)Hϕ∗HBR,kwk(n)+2Re{(wk(n))HHBR,kH(ϕ∗)Hϕ∗HBR,kwk(n)(wk−wk(n))}.

From (18), we can easily obtain that the slack variable $\lambda_{k}$ is convex with respect to $w_{k}$.

Similarly, with the feasible point $\lambda_{k}^{\left(n\right)}$, $R_{k}^{d}$ is lower bounded by (16a).

log(1+γkd)≥log(1+(λk(n))2(Ψ1,kd)(n)+Ak,i)−(λk(n))2(Ψ1,kd)(n)+Ak,i+2Re{λk(n)λk}(Ψ1,kd)(n)+Ak,i

(19) $$-\frac{{\left(\lambda_{k}^{\left(n\right)}\right)}^{2}\left(\lambda_{k}^{2}+\Psi_{1,k}^{d}+A_{k,i}\right)}{\left({\left(\Psi_{1,k}^{d}\right)}^{\left(n\right)}+A_{k,i}\right)\left({\left(\Psi_{1,k}^{d}\right)}^{\left(n\right)}+A_{k,i}+{\left(\lambda_{k}^{\left(n\right)}\right)}^{2}\right)}=\log\left(1+{\tilde{\gamma }}_{k}^{d}\right),$$

where

(20) $${\left(\Psi_{1,k}^{d}\right)}^{\left(n\right)}=\sum_{k^{\prime }\in K,k^{\prime }\neq k}{\left|\phi^{*}H_{\mathrm{BR},k}w_{k^{\prime }}^{\left(n\right)}\right|}^{2}$$

and ${\tilde{\gamma }}_{k}^{d}$ is the relaxed SINR of IoT*_k_*.

It is obviously known that the right-hand side of (19) is convex about $\lambda_{k}$ due to its quadratic form. Substituting (18) into (19), we can acquire the convexity of (19) in relation to $w_{k}$.

Next, we will relax the expressions about the SINR of UL IoT devices. Similar to (17a), we bring in auxiliary variable sets {α}, $\left\{\beta_{j}\right\}$, $\left\{\omega_{j,j^{\prime }}\right\}$, $\left\{\upsilon_{j}\right\}$ to approximate, which follow the supplementary constraints.

(21a) $$\sum_{k\in K}{\left|w_{k}\right|}^{2}\leq \alpha ,$$

(21b) $${\left|u_{j}\right|}^{2}\leq \frac{\beta_{j}^{2}}{\alpha },$$

(21c) $${\left|u_{j}^{H}H_{j^{\prime },\mathrm{RB}}{\left(\phi^{*}\right)}^{H}\right|}^{2}\leq \frac{\omega_{j,j^{\prime }}^{2}}{p},$$

(21d) $$\frac{\upsilon_{j}^{2}}{p}\leq {\left|u_{j}^{H}H_{j,\mathrm{RB}}{\left(\phi^{*}\right)}^{H}\right|}^{2},$$

where α in (21a) acts on the slack of the BS power budget. Together, $\beta_{j}$ and α in (21b) restrict the modulus of the receive beamforming vector for IoT*_j_* and react on the residual SI. $\upsilon_{j}$ in (21d) and $\omega_{j,j^{\prime }}$ in (21c) determine the boundaries of the desired received signal and the interference caused by IoT*_j_*_’_, respectively.

Although (21a) is a convex second-order cone (SOC) constraint, the constraints (21b)–(21d) are non-convex. Like (18), given the feasible point $\alpha^{\left(n\right)}$, $\beta_{j}^{\left(n\right)}$, $\omega_{j,j^{\prime }}^{\left(n\right)}$ and $u_{j}^{\left(n\right)}$, the (21b)–(21d) are transformed as

(22a) |uj|2≤2Re{(βj(n))Hβj}α(n)−(βj(n))Hβjα(α(n))2,

(22b) |ujHHj′,RB(ϕ∗)H|2≤2Re{(ωj,j′(n))Hωj,j′}p−(ωj,j′(n))Hωj,j′(n)p2,

(22c) υj2p≤(uj(n))HHj,RB(ϕ∗)Huj(n)+2Re{(uj(n))Hj,RB(ϕ∗)H(uj−uj(n))}.

Obviously, we achieve the linear constraints. Substituting (21a), (22a)–(22c) into (9), we obtain the lower bound of $\gamma_{j}^{u}$.

(23) $$\gamma_{j}^{u}\geq \frac{\upsilon_{j}^{2}}{\sum_{j^{\prime }\in J,j^{\prime }\neq j}\omega_{j,j^{\prime }}^{2}+{\left\|H_{\mathrm{SI}}\right\|}^{2}\beta_{j}^{2}+\sigma_{u,j}^{2}{\left|u_{j}\right|}^{2}},$$

where ${\left\|H_{\mathrm{SI}}\right\|}^{2}\beta_{j}^{2}$ is obtained by

(24) $$\sum_{k\in K}{\left|u_{j}^{H}H_{\mathrm{SI}}^{H}w_{k}\right|}^{2}={\left|u_{j}^{H}H_{\mathrm{SI}}^{H}\right|}^{2}\sum_{k\in K}{\left|w_{k}\right|}^{2}\leq \frac{\beta_{j}^{2}}{\alpha }\cdot {\left\|H_{\mathrm{SI}}\right\|}^{2}\cdot \alpha ={\left\|H_{\mathrm{SI}}\right\|}^{2}\beta_{j}^{2}.$$

Thus, we can acquire the slacked $R_{j}^{u}$ with the additional feasible point $\upsilon_{j}^{\left(n\right)}$ similar to (19) as follows

(25) log(1+γju)≥log(1+(υj(n))2(Ψju)(n))−(υj(n))2(Ψju)(n)+2Re{υj(n)υj}(Ψju)(n)−(υj(n))2(υj2+Ψju)(Ψju)(n)((Ψju)(n)+(υj(n))2)=log(1+γ˜ju)

where

(26) $$\Psi_{j}^{u}=\sum_{j^{\prime }\in J,j^{\prime }\neq j}\omega_{j,j^{\prime }}^{2}+{\left\|H_{\mathrm{SI}}\right\|}^{2}\beta_{j}^{2}+\sigma_{u,j}^{2}{\left|u_{j}\right|}^{2}$$

and ${\tilde{\gamma }}_{j}^{u}$ is the approximate SINR of IoT*_j_*.

The right-hand side of (25) is convex with respect to $\upsilon_{j}$ and $\Psi_{j}^{u}$, respectively. Since $\upsilon_{j}$ and $\Psi_{j}^{u}$ are convex about $u_{j}$ (see (22c) and (26)), we can obtain the convexity of (25) relating to $u_{j}$.

Above all, we achieve the convex lower bound of (14a) via the SCA method, which is reformulated as

(27) $$R\left(W,U\right)\geq \;\tilde{R}\left(W,U\right)=\sum_{k\in K}\log\left(1+{\tilde{\gamma }}_{k}^{d}\right)+\sum_{j\in J}\log\left(1+{\tilde{\gamma }}_{j}^{u}\right)$$

by (19) and (25).

We now turn our attention to the non-convex constraints (14b), (14c).

Similar to (18), constraint (14b) can be rewritten as

$$R_{\mathrm{req}}^{d}\left(\Psi_{1,k}^{d}+A_{k,i}\right)\leq {\left(w_{k}^{\left(n\right)}\right)}^{H}H_{\mathrm{BR},k}^{H}{\left(\phi^{*}\right)}^{H}\phi^{*}H_{\mathrm{BR},k}w_{k}^{\left(n\right)}$$

(28) +2Re{(wk(n))HHBR,kH(ϕ∗)Hϕ∗HBR,kwk(n)(wk−wk(n))}.

Meanwhile, by applying (21a), (22a)–(22c), constraint (14c) can be expressed as

(29) $$\sqrt{R_{\mathrm{req}}^{u}}|[\left\|H_{\mathrm{SI}}\right\|\beta_{1},\ldots ,\left\|H_{\mathrm{SI}}\right\|\beta_{J},\omega_{j,1},\ldots ,\omega_{j,j-1},\omega_{j,j+1},\ldots ,\omega_{j,J},\sigma_{u,j}u_{j}^{H}]|\leq \upsilon_{j}.$$

Therefore, we have obtained the linear constraint of (28) and SOC constraint of (29) to approximate (14b) and (14c), respectively.

Finally, the problem 𝒫2 is reconstructed as

(30) $${𝒫2}^{\prime }:\underset{W,U}{\max}\;\tilde{R}\left(W,U\right)$$

s.t. (13b), (13c), (28), (29).

The problem ${𝒫2}^{\prime }$ is a convex SCA that can be optimally solved via a convex optimization tool such as CVX.

The proposed SCA algorithm for solving subproblem one is summarized in Algorithm 1.
**Algorithm 1:** Proposed SCA Algorithm for Problem 𝒫2
1$\mathrm{Initialization}:\;\phi^{*},\;\{w_{k}^{\left(0\right)}\},\;\{u_{j}^{\left(0\right)}\},\;\{\lambda_{k}^{\left(0\right)}\},\;\{\alpha^{\left(0\right)}\},\;\{\beta_{j}^{\left(0\right)}\},\;\{\omega_{j,j^{\prime }}^{\left(0\right)}\},\;\{\upsilon_{j}^{\left(0\right)}\}.$2**Repeat:**3$\mathrm{Calculate}\;\{w_{k}\},\;\{u_{j}\},\;\{\lambda_{k}\},\;\{\alpha \},\;\{\beta_{j}\},\;\{\omega_{j,j^{\prime }}\},\;\{\upsilon_{j}\}\;\mathrm{by}\;\mathrm{using}\;\mathrm{CVX}\;\mathrm{through}\;{𝒫2}^{\prime }.$4$\mathrm{Update}\;w_{k}^{\left(n\right)}=w_{k}^{*},\;u_{j}^{\left(n\right)}=u_{j}^{*},\;\lambda_{k}^{\left(n\right)}=\lambda_{k}^{*},\;\alpha^{\left(n\right)}=\alpha^{*},\;\beta_{j}^{\left(n\right)}=\beta_{j}^{*},\;\omega_{j,j^{\prime }}^{\left(n\right)}=\omega_{j,j^{\prime }}^{*},\;\upsilon_{j}^{\left(n\right)}=\upsilon_{j}^{*}.$5Set n=n+1.6**Until:** The value of sum SE converges.7$\mathrm{Output}:\;w^{*},\;U^{*}.$


### 3.2. RIS Phase Shifts Design

We now focus on the optimization of ϕ when the beamforming matrices are fixed. The problem 𝒫1 is simplified as

(31a) $$𝒫3:\underset{\phi }{\max}\sum_{k\in K}\log\left(1+\frac{{\left|\phi H_{\mathrm{BR},k}w_{k}^{*}\right|}^{2}}{\Psi_{1,k}^{d}+A_{k,i}}\right)+\sum_{j\in J}\log\left(1+\frac{p{\left|{\left(u_{j}^{*}\right)}^{H}H_{j,\mathrm{RB}}\phi^{H}\right|}^{2}}{\Psi_{1,j}^{u}+\Psi_{\mathrm{SI},j}+\sigma_{u,j}^{2}{\left|u_{j}^{*}\right|}^{2}}\right)$$

(31b) $$s.t.\;R_{k}^{d}\left(\phi \right)\geq R_{\mathrm{req}}^{d},\forall k\in K,$$

(31c) $$R_{j}^{u}\left(\phi \right)\geq R_{\mathrm{req}}^{u},\forall j\in J,$$

(13b), (13d).

The variable ϕ in the non-convex objective function (31a) with fractional structure is associated with DL and UL IoT devices. Moreover, 𝒫3 has an additional unit-modulus constraint. Especially in scenario two, $A_{k,i}$ is also relevant to ϕ (see (15)). All the mentioned properties determine that 𝒫3 is more challenging compared with 𝒫2.

Since the DRL method is implemented without slackness, it can reduce the optimal performance loss and computational complexity. Based on a model-free way, DRL can be directly used to solve tough mathematical problems. Therefore, scholars resort to the DRL method as a powerful tool to tackle wireless network issues [40,41]. In addition, it works with non-labeled data sets that merit high storage efficiency [42].

SAC is a model-free DRL algorithm based on maximum entropy, which avoids local optimization via entropy regularization. Meanwhile, it applies two independent *Q* networks and an off-policy scheme to promote stability and learning efficiency [43], which is also suitable for discrete action space [44].

#### 3.2.1. Markov Decision Process

Considering that the Markov decision process (MDP) is the theoretical cornerstone of reinforcement learning (RL) [45], we assume that the RIS controller exercises an agent role for pursuing the maximum reward through sequential decision making, thus maximizing the sum SE. Hence, we map the RIS-aided FD system with the key elements of MDP as follows.

- Action: A phase shifts vector is treated as a one-dimensional discrete action, such as an action at time step *t* defined as
  (32) $$a_{t}≜[\phi_{t}(1),\phi_{t}(2),\ldots ,\phi_{t}(L)].$$
  We normalize the actions via the policy network to meet the constraint (13d). It is worthwhile mentioning that the action is only relevant to the phase shifts with *L* elements, which improves the learning efficiency in the multi-user scenario through a reduced action space.

- State: The state vector includes the SINR of each IoT device and is represented as
  (33) $$s_{t}≜[\gamma_{1,t}^{d},\gamma_{2,t}^{d},\ldots ,\gamma_{K,t}^{d},\gamma_{1,t}^{u},\gamma_{2,t}^{u},\ldots ,\gamma_{J,t}^{u}]$$
  at time step *t*. $s_{t}$ can be acquired via the interaction between the agent RIS controller and the environment based on the given phase shifts and state of the previous time step. Given that the characteristic dimension of the state can reduce the RL performance [46], the state vector should be whitened each time after the SINR is observed. Since the state is associated with each IoT device’s wireless communication condition, it can efficiently cover the multi-user scenario. In general, the phase shifts vector to be performed at the current step only depends on the real-time conditions of each IoT device without regard to the previous action, which simplifies the interaction.

- Reward: Considering that the state is only dependent on each individual instead of the entirety, we take the reward as a guide of the global policy to the RIS controller. With the aim of the maximum sum SE, we choose a modified objective as a reward.
  (34) $$r_{t}≜\{\begin{array}{l} \sum_{k\in K}R_{k,t}^{d}+\sum_{j\in J}R_{j,t}^{u},\forall R_{k,t}^{d},R_{j,t}^{u}\geq R_{\mathrm{req}}^{d},R_{\mathrm{req}}^{u},\\ 0,\;\mathrm{otherwise}, \end{array}$$
  where $R_{k,t}^{d}$ and $R_{j,t}^{u}$ indicate the return of SE at time step *t* for IoT*_k_* and IoT*_j_*, respectively. Notably, we bring in the penalty to inspire the agent to find a policy that satisfies the QoS constraints (31b), (31c).

**Figure 2:**
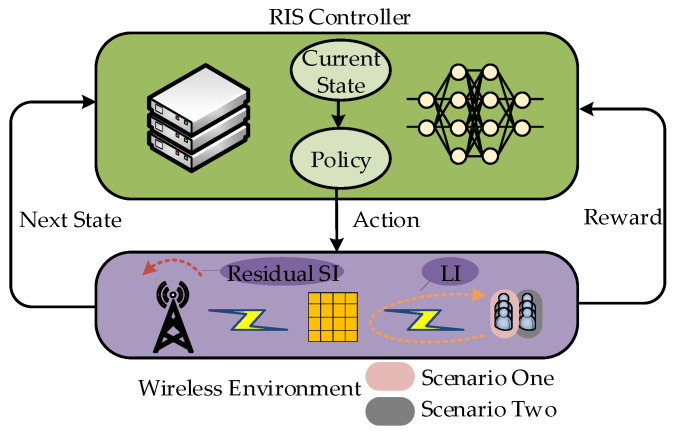
An MDP of RIS controller and environment.

In summary, when the RIS controller chooses a series of phase shifts with a certain probability, the SINR of each IoT device will be updated. Thus, the next state is transitioned with the corresponding reward acquired. The process of the RIS controller interacting with the defined wireless environment model (i.e., the urban outdoor scenario one or two) can be deemed as an MDP, visualized in Figure 2. It is noteworthy that Scenario One and Scenario Two in Figure 2 only differ in wireless environments where the IoT devices are located. This distinction will not influence the workflow of the model-free DRL method due to the interaction mechanism between the agent and the environment.

The RIS controller decision making would introduce delay in practice, which takes seconds due to the computational power of the RIS controller and the magnitude of changes in the environment. Nevertheless, if the RIS controller has been saturated from learning, it will immediately make an optimal decision at the general channel condition changes. More importantly, the RIS controller can also rapidly cope with large environmental transformations over time. This is because the RIS controller would learn more about potential fluctuations of the environment in the long term, which endows the RIS controller with the capability to tackle extreme cases easily. Overall, the DRL-based method can better reflect advantage in the long run.

#### 3.2.2. Mechanism of Soft Actor–Critic Learning

The SAC architecture contains actor and critic networks for action selection and evaluation. Specifically, a random policy network constitutes the actor network. In addition, the critic network consists of online and target subnetworks, which include two online and two target *Q* networks, respectively. The online and target *Q* networks have the same structure but differ in the update method and frequency. The critic network copes with the provided action from the actor network and selects a minimum *Q* value from the two calculated soft *Q* values, thus avoiding overfitting [47]. Our goal is to train the above SAC network to acquire the ability to output the best phase shifts vector over extended interactions with the environment. The training of the SAC network, including implementation and learning processes, is described in detail.

- Implementation: $s_{t+1}$ and $r_{t}$ are derived correspondingly by inputting $a_{t}$ at the current state $s_{t}$. Then, the acquired transition tuple $\left(s_{t},a_{t},r_{t},s_{t+1}\right)$ is stored in a replay buffer that gradually enriches with multiple interactions. Notably, for the sake of the agent to explore comprehensively, the replay buffer can be stuffed off-policy.
- Learning: In each time step, a mini-batch containing several transition tuples is randomly sampled from the replay buffer. Then, the learning process in a mini-batch is as follows.

In particular, unlike the conventional DRL-based method, the soft-state value function in the SAC algorithm introducing a relative entropy is defined as

(35) $$V\left(s_{t};\theta_{i}\right)=\pi_{\varphi }{\left(s_{t}\right)}^{T}\left[Q(s_{t},a;\theta_{i})-\alpha \log\left(\pi (s_{t})\right)\right],$$

where $\theta_{i}$(i∈{1,2}) is the network parameter relating to the *i*-th online *Q* network. $\pi_{\varphi }(s_{t})\in {\left[0,1\right]}^{\left|A\right|}$ represents the policy with probability [0,1], calculated through the policy network, in the action space |A|. φ and α are the parameters with respect to the actor network and temperature. α also determines the importance of entropy relative to the *Q* value. In addition, the *Q* value is an action-state value function written as

(36) $$Q(s_{t},a;\theta_{i})=r(s_{t},a)+\gamma E_{s_{t+1}∼p(s_{t},a)}\left[V\left(s_{t+1};\theta_{i}\right)\right],$$

where γ is the discount factor used to calculate the cumulative returns. $p(s_{t},a)$ indicates the probability of executing a specific action. Accordingly, the state is transitioning from $s_{t}$ to $s_{t+1}$. Above all, the SAC algorithm intends to explore as many varied actions as possible to maximize the target entropy based on a given $s_{t}$. This process is also accompanied sequentially by loss calculation and parameter updating for the three modules below.

First, the online *Q* network is trained by minimizing the Bellman residual as follows.

(37a) $$\theta_{i}\leftarrow \theta_{i}-\lambda_{Q}\nabla_{\theta_{i}}J_{Q}\left(\theta_{i}\right),$$

(37b) $$J_{Q}\left(\theta_{i}\right)=E_{s_{t}∼D}\left[\frac{1}{2}{\left(Q(s_{t},a;\theta_{i})-Q(s_{t},a;\theta_{i}^{-})\right)}^{2}\right]$$

where $\lambda_{Q}$ and $J_{Q}\left(\theta_{i}\right)$ represent the *Q* network’s learning rate and loss function. $\theta_{i}^{-}$ is the network parameter concerning the *i*-th target *Q* network. *D* denotes the replay buffer.

Similar to the critic network, the actor network is trained subsequently.

(38a) $$\varphi \leftarrow \varphi -\lambda_{\pi }\nabla_{\varphi }J_{\pi }\left(\varphi \right),$$

(38b) $$J_{\pi }\left(\varphi \right)=E_{s_{t}∼D}\left[\pi_{\varphi }{\left(s_{t}\right)}^{T}\left[\alpha \log\left(\pi_{\varphi }(s_{t})\right)-Q(s_{t},a;\theta )\right]\right],$$

where $\lambda_{\pi }$ denotes the actor network’s learning rate. $J_{\pi }\left(\varphi \right)$ signifies the loss function of the policy network.

Meanwhile, α is also trained by updating the loss function to avoid being set as a hyper-parameter and to reduce the estimation error, written as

(39a) $$\alpha \leftarrow \alpha -\lambda \nabla_{\alpha }J\left(\alpha \right),$$

(39b) $$J\left(\alpha \right)=\pi_{\varphi }{\left(s_{t}\right)}^{T}\left[-\alpha \left(\log\left(\pi_{\varphi }(s_{t})\right)+\bar{H}\right)\right],$$

where λ represents the temperature’s learning rate. J(α) indicates the loss of temperature. $\bar{H}$ is a constant vector equivalent to the hyper-parameter of target entropy.

Finally, the two target *Q* network parameters are updated as

(40) $$\theta_{i}^{-}\leftarrow \rho \theta_{i}+\left(1-\rho \right)\theta_{i}^{-},$$

where ρ is the soft update factor.

The stage of (37a)–(40) implies the agent learning in one time step. After the *ET* time steps of *E* episodes, the agent’s performance will saturate, and the optimized phase shifts vector $\phi^{*}$ has been acquired.

**Figure 3:**
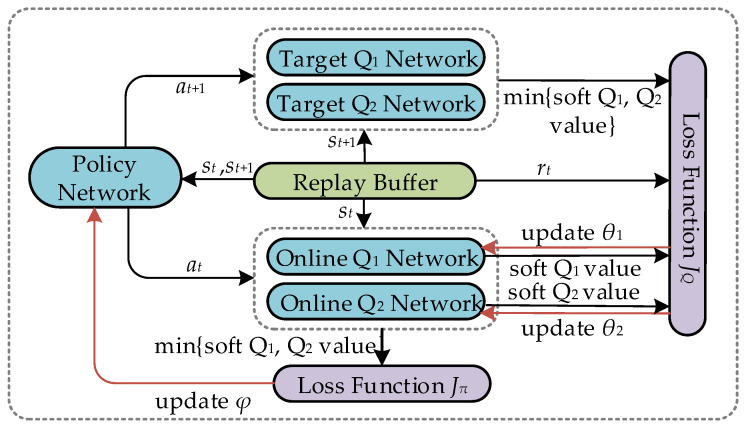
Framework of the SAC.

Figure 3 shows the parameter updating process for actor and critic networks, and the proposed SAC algorithm is summarized in Algorithm 2.
**Algorithm 2:** Proposed SAC Algorithm for Problem 𝒫3
1$\mathrm{Initialization}:\;w^{*},\;U^{*},\;\theta_{1},\;\theta_{2},\;\theta_{1}^{-},\;\theta_{2}^{-},\;\varphi ,\;\alpha ,\;D.$2Set e=1.3**Repeat:**4Set t=1.5$\mathrm{Receive}\;\mathrm{initial}\;\mathrm{observation}\;\mathrm{state}\;s_{t}.$6**Repeat:**7$\mathrm{Feed}\;a_{t}\;\mathrm{the}\;\mathrm{phase}\;\mathrm{shifts}\;\mathrm{vector}\;\mathrm{to}\;\mathrm{the}\;\mathrm{environment}\;\mathrm{with}\;\mathrm{the}\;\mathrm{obtained}\;\mathrm{CSI}\;\mathrm{and}\;\mathrm{the}\;\mathrm{given}\;w^{*},\;U^{*}\;\mathrm{to}\;\mathrm{calculate}\;\mathrm{the}\;\mathrm{next}\;\mathrm{state}\;s_{t+1}\;\mathrm{and}\;\mathrm{reward}\;r_{t}$ by (32)–(34).
8$\mathrm{Store}\;\mathrm{the}\;\mathrm{transition}\;\mathrm{tuple}\;(s_{t},a_{t},r_{t},s_{t+1}).$ in the replay buffer *D*.9Randomly sample min-batch transition tuples with batch size *N* from *D*.10$\mathrm{Update}\;\mathrm{the}\;\mathrm{parameters}\;\theta_{1}\;\mathrm{and}\;\theta_{2}$ of the online *Q* networks by (37a), (37b).11Update the parameter φ. of the actor *Q* networks by (38a), (38b).12Update the temperature parameter α. by (39a), (39b).13$\mathrm{Update}\;\mathrm{the}\;\mathrm{parameters}\;\theta_{1}^{-}\;\mathrm{and}\;\theta_{2}^{-}.$ of the target *Q* networks by (40).14Set t=t+1.15Until: t>T.16Set e=e+1.17Until: e>E.18$\mathrm{Output}:\;\phi^{*}.$


#### 3.2.3. Proposed Deep Neural Network Design

Each NN contains an input, an output, and two hidden layers. The hidden layer of both the actor and critic networks includes $L_{i}$(i∈{1,2}) neurons. The input and output layers of the actor network have (J+K) and *L* neurons—the same number as state and action sizes, respectively. Since the input layer of the critic network additionally concatenates the action that the actor network selects, it includes (J+K+L) neurons. After getting the action and state, the critic network evaluates the action and gives a corresponding *Q* value as an assessment result, thus occupying one neuron at its output layer. The ReLU activation functions are adopted after the hidden layers of actor and critic networks. Moreover, the output layer of the critic network applies the Linear activation function. In contrast, the output layer of the actor network introduces a Softmax activation function to ensure that the discrete actions are distributed with effective probabilities. All networks use an Adam optimizer for parameter updating.

### 3.3. Algorithm Development and Computational Complexity

The proposed two-step algorithm is presented in Algorithm 3 by merging the two solutions for ${𝒫2}^{\prime }$ and 𝒫3. In particular, ${𝒫2}^{\prime }$ is a convex problem that ensures the convergence in each iterative sub-solution. Meanwhile, the convergence of 𝒫3 is also guaranteed by tuning the hyper-parameters. Since each sub-solution of ${𝒫2}^{\prime }$ and 𝒫3 outputs the optimal value, the SE value after each iteration *m* satisfies ${\mathrm{SE}}^{\left(m+1\right)}\geq {\mathrm{SE}}^{\left(m\right)}$. Moreover, the objective SE is upper-bounded depending on the transmit power constraint and the interference level, so the convergence of Algorithm 3 can be acquired.
**Algorithm 3:** Proposed Two-step Algorithm for Problem 𝒫1
1$\mathrm{Initialization}:\;\phi^{\left(0\right)},\;w^{\left(0\right)},\;U^{\left(0\right)}.$2**Repeat:**3$\mathrm{Solve}\;{𝒫2}^{\prime }\;\mathrm{with}\;\mathrm{fixed}\;\phi^{\left(m\right)}$ by Algorithm 1.4$\mathrm{Update}\;w^{\left(m\right)}=w^{*},\;U^{\left(m\right)}=U^{*}.$5$\mathrm{Solve}\;𝒫3\;\mathrm{with}\;\mathrm{fixed}\;w^{\left(m\right)},\;U^{\left(m\right)}$ by Algorithm 2.6$\mathrm{Update}\;\phi^{\left(m\right)}=\phi^{*}.$7Set m=m+1.8**Until:** The value of sum SE converges.9$\mathrm{Output}:\;w^{*},\;U^{*},\;\phi^{*}.$


Since ${𝒫2}^{\prime }$ only contains linear and SOC constraints, the computational complexity of Algorithm 1 is relatively low. The polynomial time complexity considers $O({\left(J+K+2\right)}^{2.5}$${\left(KN_{t}+JN_{r}+J^{2}+J+K\right)}^{2}+{\left(J+K+2\right)}^{3.5})$ [48]. According to the dimensions of the aforementioned NN, the complexity of Algorithm 2 is $O(2((J+K+L)L_{1}$$+L_{1}L_{2}+L_{2})+(J+K)$$L_{1}$$+L_{1}L_{2}+L_{2}L)$. Compared to the fully exhaustive search method with exponential complexity applied in a combinatorial problem 𝒫3, the complexity of Algorithm 2 is significantly reduced.

## 4. Performance Evaluation

This section provides comprehensive numerical results to validate the effectiveness of our proposal. The two-step solution is solved through Matlab and Python tools. To be specific, subproblem one with parameters W and U is worked out through Matlab (version: Matlab R2020b), and subproblem two with parameter ϕ is tackled with Python (version: Python 3.8).

### 4.1. Simulation Setup and Parameters Setting

**Figure 4:**
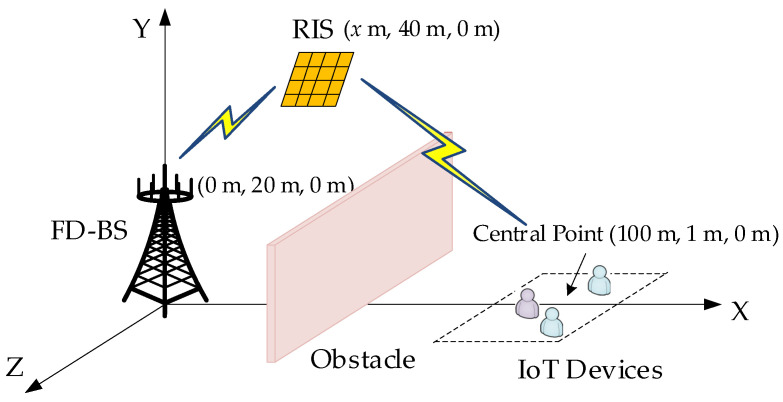
Simulation setup.

According to Figure 4, as the simplified system model, we consider a three-dimensional coordinate system where the BS antennas and RIS are located at (0 m, 20 m, 0 m) and (*x* m, 40 m, 0 m), respectively. In addition, the IoT devices are uniformly distributed in a horizontal square region with a side length of 40 m, which is centered at (100 m, 1 m, 0 m), where 1 m signifies the working height of each IoT device. Without loss of generality, for J=K=3, we set the coordinates of the DL IoT devices as (90 m, 1 m, 10 m), (100 m, 1 m, −10 m), and (110 m, 1 m, 10 m), while UL IoT devices as (90 m, 1 m, −10 m), (100 m, 1 m, 10m), and (110 m, 1 m, −10 m). The QoS of each IoT device is considered 1 bps/Hz to guarantee all devices, especially the devices with relatively poor channel conditions, in normal communications. The channel path loss of large-scale fading is defined as

(41) PL=−35.6−22lg(d),

where *d* represents the distance between two nodes. The value of −22 indicates that the path loss exponent is set at 2.2. Meanwhile, the value of −35.6 denotes the path loss at the reference distance of 1 m, which depends on the average channel attenuation and antenna characteristics [49].

The small-scale fading is modeled by

(42a) $$H=\sqrt{\frac{\varepsilon }{1+\varepsilon }}H^{LoS}+\sqrt{\frac{1}{1+\varepsilon }}H^{NLoS},$$

(42b) $$h=\sqrt{\frac{\varepsilon }{1+\varepsilon }}h^{LoS}+\sqrt{\frac{1}{1+\varepsilon }}h^{NLoS},$$

where $H^{LoS}$ and $h^{LoS}$ represent the deterministic LoS components of BS-RIS and RIS-IoT device channels for UL/DL, respectively. $H^{NLoS}$ and $h^{NLoS}$ mean the stochastic NLoS components in a similar manner [50]. The rician factor ε is set to 10. We assume the AWGN $\sigma_{d}^{2}=$$\sigma_{u}^{2}=$−107 dBm and the equivalent AWGN $\sigma_{\mathrm{UDI},k}^{2}=-107$ dBm, $\sigma_{\mathrm{LI},k}^{2}=-96$ dBm (Since we assume LI in scenario one is an approximated AWGN, we set the value of $\sigma_{\mathrm{LI},k}^{2}$ −96 dBm at x=50 m according to the average distance between the RIS and the IoT to manifest its subordinate influence compared with the UDI in this occasion). Considering the different channel characteristics and the up-to-date capability of SI elimination, we set the rician factor *a* and power elimination level $\sigma_{\mathrm{SI}}^{2}$ of the residual SI at BS to 1 and −100 dB, respectively [17]. Other required simulation parameters will be listed in the title of the corresponding figures. Each hidden layer of our proposed SAC algorithm contains 256 neurons. The main DRL-related hyper-parameters refer to Table 2.

Furthermore, we introduce a fully exhaustive search (i.e., an upper bound for discrete phase shifts) method as a benchmark to solve 𝒫3. However, due to the non-deterministic polynomial time, the exhaustive search approach lacks practicality in real-world applications. We additionally take a relatively low complexity local fixed-point iteration method [51], where the complexity is $O({\left(L+1\right)}^{2}(K^{2}+J^{2}))$ and $O({\left(L+1\right)}^{2}(K^{2}+J^{2}+$KJ)) for scenarios one and two, respectively. Meanwhile, to highlight the gain from the phase shifts optimization, we additionally bring the random phase shifts method and take the case without RIS as a reference. Finally, the Riemannian manifold under continuous phase shifts is also introduced as an ideal case to reveal the effectiveness of the discrete phase shifts methods [37]. The simulation results are averaged based on 300 channel realizations.

### 4.2. Optimizer Performance

**Figure 5:**
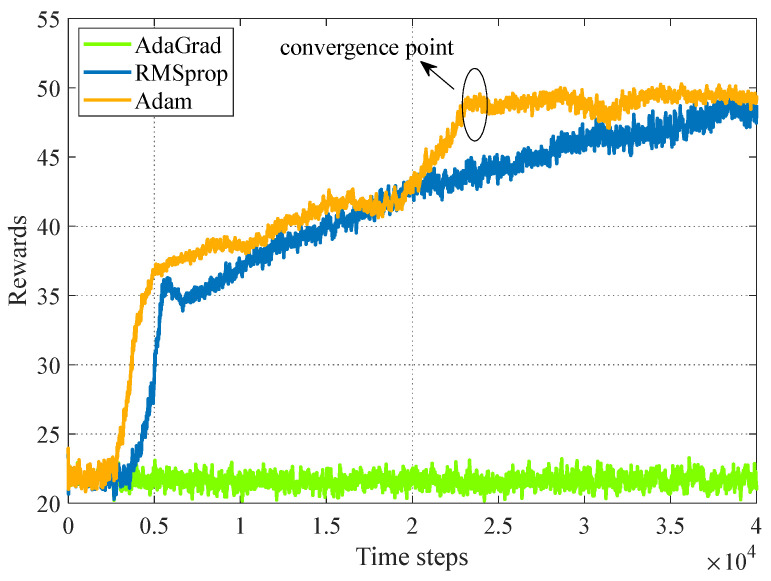
Optimizer performance. System parameters: $N_{t}=N_{r}=J=K=8$, b=4, L=16, P=30 dBm, p=20 dBm, x=0 m in scenario one.

AdaGrad, RMSprop, and Adam all have their own merits. However, empirical results demonstrate that Adam works well in practice and compares favorably to other stochastic optimization methods [52]. In this regard, we take Adam optimizer in the SAC network and give a performance comparison between AdaGrad, RMSprop, and Adam based on our framework. From Figure 5, the learning process of the Adam optimizer saturates faster than RMSprop, while the AdaGrad optimizer is absolutely unable to work in our framework.

### 4.3. Convergence of Algorithm 3

**Figure 6:**
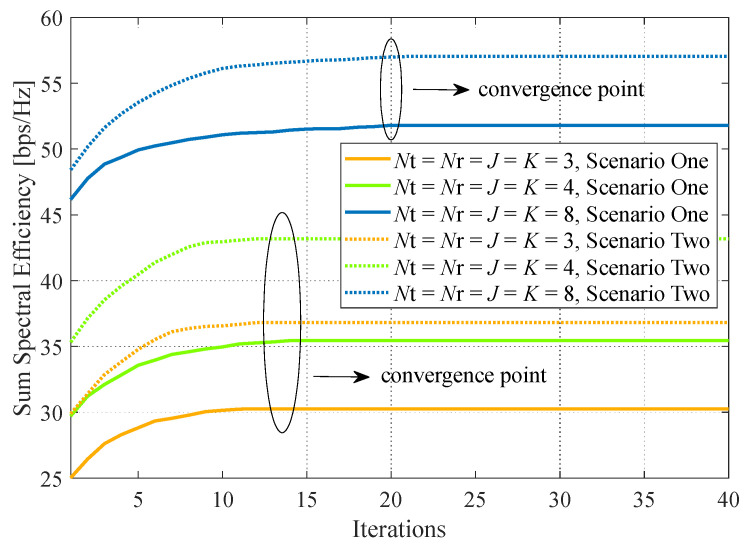
Convergence performance. System parameters: b=4, L=16, P=30 dBm, p=20 dBm, x=0 m.

Figure 6 shows the convergence behavior of the proposed two-step algorithm with respect to different parameter settings in both scenarios. It can be observed that increasing the number of BS antennas and IoT devices with the fixed RIS configuration will slow down the convergence. For instance, the parameter settings of $N_{t}=N_{r}=J=K=3$ and $N_{t}=N_{r}=$J=K=4 undergo 10–15 iterations to convergence, while nearly 20 iterations are required for setting $N_{t}=N_{r}=J=K=8$. Moreover, there are no evident distinctions for convergence between the two scenarios. It confirms that the proposed DRL method is less affected by the scenarios.

### 4.4. Impact of the RIS Location

**Figure 7:**
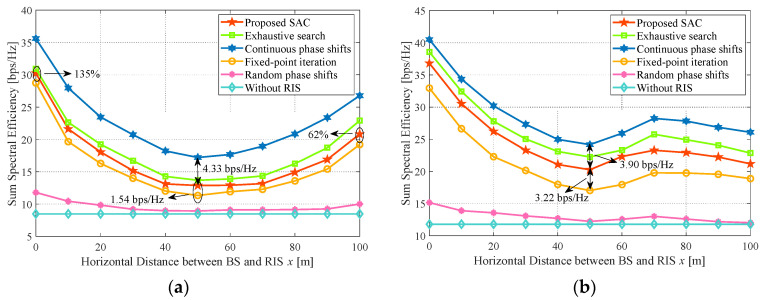
Impact of the horizontal distance between BS and RIS. System parameters: $N_{t}=N_{r}=$ J=K=3, b=4, L=16, P=30 dBm,p=20 dBm. (**a**) Sum SE versus *x* in scenario one; (**b**) Sum SE versus *x* in scenario two.

Figure 7a illustrates the performance impact of RIS location in scenario one. It is evident that the RIS deployed either close to the BS or the IoT devices will improve the sum SE. The proposed algorithm can obtain 135% and 62% performance gains with RIS deployment on the BS (x=0 m) and IoT devices side (x=100 m) compared with that at x=50 m, respectively. The reason is that RIS can reconstruct the signal utmost at these positions, thus significantly enhancing the desired signal and reducing interference via adjusting the different transmission directions when the LoS link is severely blocked. Further, with *x*(0<x≤50 m) increasing, the sum SE decreases. It is caused by the weakening reflected signal, which degrades the capability of suppressing the residual SI at BS. When the deployment of RIS is far from the BS and begins approaching the IoT devices, the sum SE increases. It implies that the RIS next to the IoT devices can further alleviate the UDI. In addition, it is found that the continuous phase shifts method outperforms other algorithms owing to the infinite RIS resolution. Although the proposal’s performance is slightly lower than the exhaustive search method, it is better than the sub-optimal local fixed-point iteration method. This is because the fixed-point iteration method easily falls into local optimization when RIS includes many reflection elements. Next, because of the aimless signal reconstruction of the random phase shifts method, it achieves trivial profit from the RIS and is slightly better than the case without RIS. It is also for this reason that the random phase shifts method, or the case without RIS, is naturally less (≤2.84 bps/Hz) or none affected by the location of the RIS.

Figure 7b shows the impact of the RIS location in scenario two. Since the LI on the IoT devices side is emphasized in this scenario, it will further highlight the deployment benefit when RIS is located on the BS side. That is because we assume BS can absolutely eliminate the LI on the BS side, while the IoT devices incur the major performance impact of LI at the consideration of the trivial UDI. The trend of the curves is also consistent with Figure 7a when *x* is less than 70 m, which is the same reason as Figure 7a has explained. For x>70 m, the sum SE decreases with *x* increasing. It is caused by the enhanced LI when RIS is excessively approaching the IoT devices side that the loss of LI will partially offset the profit of signal reconstruction.

Furthermore, the performance gap between the proposal and the fixed-point iteration method is distinct in scenario two, where the gap at x=50 m is 3.22 bps/Hz, compared to 1.54 bps/Hz at the same location in scenario one. It is relevant to the different objective functions in relation to phase shifts in each scenario. Specifically, the related objective in scenario two is more complicated than that in scenario one, leading to a larger error of the fixed-point iteration method when calculating the unit operations of each related phase shift to the next iteration. However, the proposed algorithm is based on the model-free, which does not particularly care about the concrete objective structure. Similarly to the fixed-point iteration method, the continuous phase shifts scheme based on the Riemannian manifold tends to deviate from manifold space when updating the tangent space in scenario two. Thus, the gap between the continuous phase shift and the proposal is smaller in scenario two than in one, such as the 3.90 bps/Hz performance gap at x=50 m in Figure 7b, while 4.33 bps/Hz in Figure 7a. Even if under the heavy LI at x=100 m of scenario two, the proposed algorithm outperforms the continuous phase shifts and fixed-point iteration methods by 1.03 bps/Hz and 0.68 bps/Hz relative gains with respect to scenario one, respectively. It suggests that the proposed algorithm is more advantageous in LI mitigation than the other algorithms under the scenario handoff.

Notably, scenario two with LI stressed is more likely to reproduce due to the high density of population and buildings in the smart cities. Thus, our proposal is more adaptable to practical application in the urban outdoor environment.

Joining Figure 7a,b, we can conclude that the SE performance concerning RIS mainly depends on RIS location and the wireless environment of IoT devices. In any case, the performance gain from signal reconstruction of the optimized RIS is better than that of non-RIS and random RIS methods. Since the varied performance among scenarios mainly depends on the two relevant factors we have discussed above, we only display the numerical results with respect to other influencing elements (as we shall see below) at x=50 m in scenario two. We can concisely highlight our proposal through the scenario handoff from scenarios one to two and the trade-off of interference mitigation between the residual SI and LI at x=50 m.

### 4.5. Impact of the Number of RIS Reflection Elements

**Figure 8:**
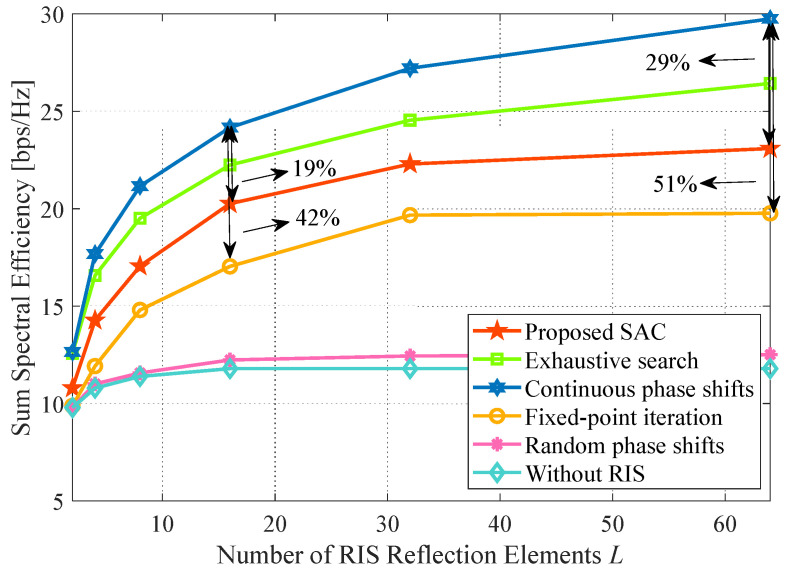
Impact of the number of RIS reflection elements. System parameters: $N_{t}=N_{r}=J=K=3$, b=4, P=30 dBm, p=20 dBm, x=50 m.

Figure 8 shows the influence of the number of RIS reflection elements. We can observe that the sum SE increases, and the gap between the continuous phase shifts method and others widens with the increase in *L*. This expected result is mainly produced by the more reflection elements, the more cumulative gain from the reconstructed signal. For L=64, the fixed-point iteration and proposed algorithms are obviously weaker than the continuous phase shifts method. The discrepancy in performance loss of the proposed algorithm ascends from 19% at L=16 to 29% at L=64 in reference to the continuous phase shifts method. Same as this, the fixed-point iteration method is up from 42% to 51%. The reason is that our proposal reduces computational complexity at the cost of a certain performance when the action dimension is large. Moreover, the fixed-point iteration easily falls into the local optimization as the number of *L* is large, which is consistent with the conclusion in Figure 7a.

### 4.6. Impact of the Number of Bits

**Figure 9:**
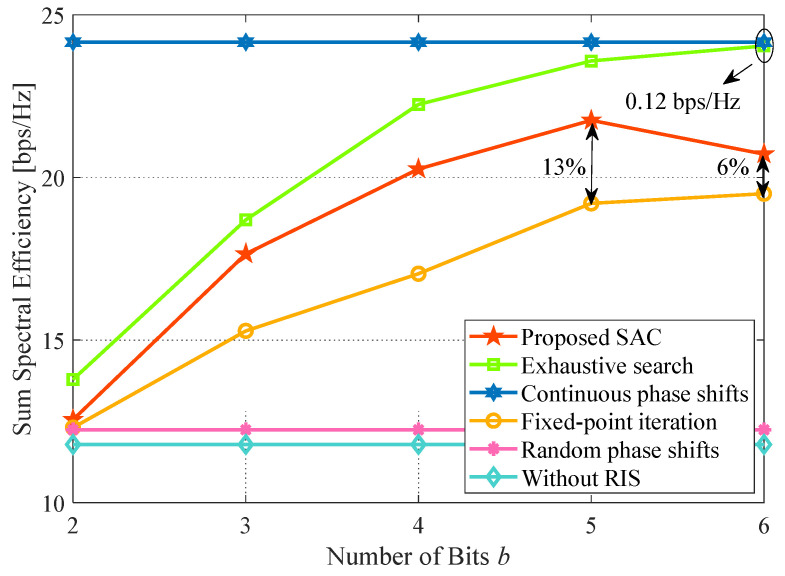
Impact of the number of RIS resolution bits. System parameters: $N_{t}=N_{r}=J=K=3$, L=16, P=30 dBm, p=20 dBm, x=50 m.

In Figure 9, we evaluate the performance trend under different *b*. It can be seen that the performance improves with *b* (b≤5) increases except for the resolution uncorrelated methods, such as the continuous phase shifts, random phase shifts, and case without RIS methods. Especially for the random phase shifts method, the gain of RIS only depends on the number of reflection elements instead of the resolution. It accounts for the fact that different random phases in one element cannot reconstruct the signal well due to the casual transmission direction. Additionally, the performance of the exhaustive search method is approximately equal to the continuous phase shifts method when *b* is set to 6 bits and only loses 0.12 bps/Hz. It explains that the discrete phase shifts method can also approach the continuous one at some point. Therefore, the rationality of our proposal adopting discrete phase shifts is proved.

Nevertheless, the performance of the proposed algorithm decreases at 6 bits. Compared with the fixed-point iteration method, the proposal has an extra 7% gain deficit at b=6 relative to b=5. This is due to the fact that a larger resolution will incur an exponential increment in the action space, which will reduce the learning efficiency. It suggests that our proposal should find a trade-off between performance and resolution.

### 4.7. Impact of the Residual Self-Interference

**Figure 10:**
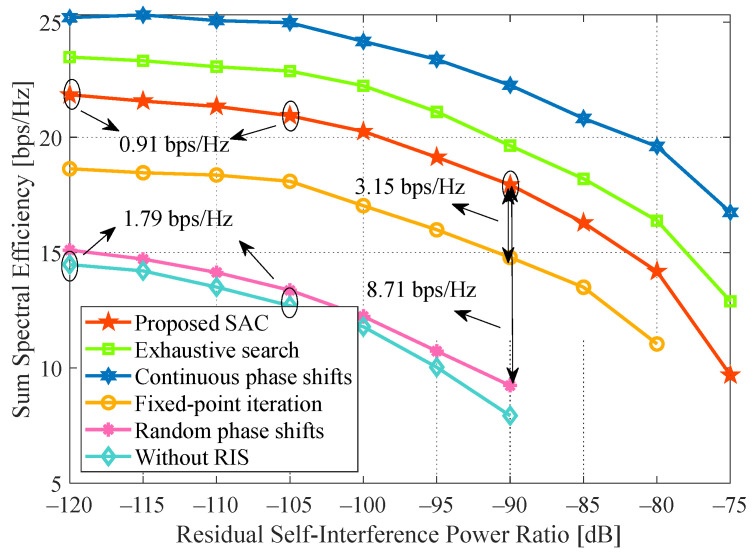
Impact of the level of residual self-interference. System parameters: $N_{t}=N_{r}=J=K=3$, b=4, L=16, P=30 dBm, p=20 dBm, x=50 m.

Figure 10 presents the sum SE trend under different residual SI levels. Obviously, the performance is more afflicted with the increased residual SI, and our proposal can better eliminate the residual SI at the condition of low (−120 dB $\leq \sigma_{\mathrm{SI}}^{2}\leq -105$ dB) and normal (−105 dB $\leq \sigma_{\mathrm{SI}}^{2}\leq -90$ dB) levels. To be specific, the maximum loss of our proposal caused by the increasing residual SI is 0.91 bps/Hz and 3.01 bps/Hz during low and normal levels, respectively, whereas that of the case without RIS is 1.79 bps/Hz and 4.77 bps/Hz. For the ideal case, the corresponding loss of the continuous phase method is 0.22 bps/Hz and 2.71 bps/Hz. It demonstrates that it is practical to adopt the proposed algorithm to reduce the FD performance loss by the residual SI.

The sum SE drops distinctly when the residual SI level exceeds −90 dB. At $\sigma_{\mathrm{SI}}^{2}=-90$ dB, our proposal outperforms the fixed-point iteration and random phase shifts methods by 3.15 bps/Hz and 8.71 bps/Hz, respectively. Neglecting the benefit factor of the optimized RIS by comparing the random phase shifts method, we can infer that the proposed algorithm can also brilliantly restrain the LI to improve performance.

### 4.8. Impact of the Transmit Power

**Figure 11:**
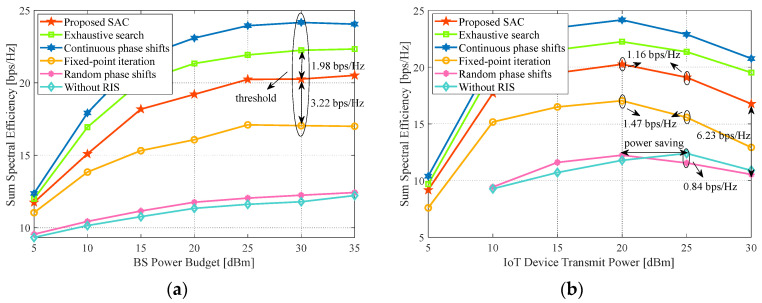
Impact of the transmit power. System parameters: $N_{t}=N_{r}=J=K=3$, b=4, L=16, x=50 m. (**a**) Sum SE versus *P*; (**b**) Sum SE versus *p*.

Figure 11a,b show the performance impact of the transmit power. In Figure 11a, with the BS power budget increasing, the sum SE improves and reaches the bottleneck at 30 dBm. This is mainly due to the effect of residual SI and DL to DL interference. For instance, the excess transmit power at BS will severely enhance its SI, thus degrading the performance. Accordingly, the actual power allocated by BS will be lower than the threshold to seek the optimum sum SE when the transmit power budget is oversaturated. At the saturation point, the proposed algorithm outweighs the fixed-point iteration method by 3.22 bps/Hz, while it only has 1.98 bps/Hz SE less than the exhaustive search method.

On the other hand, we further compare the influence of the IoT device transmit power. The simulation result in Figure 11b presents that performance starts to decline when the transmit power of IoT devices is over 20 dBm, except for the case without RIS. For example, the proposal and fixed-point iteration methods degrade 1.16 bps/Hz and 1.47 bps/Hz from p=20 dBm to 25 dBm, respectively. We can infer that the composite strong LI and UL to UL interference due to the superfluous transmit power mainly creates the trend of these curves.

Because of no LI in the case without RIS, the peak rests on p=25 dBm. However, if *p* keeps rising, the performance decreases due to the difficulty of BS in decoding the signal mixed with an intensive UL to UL interference. It explains that the IoT device transmit power in real-world applications should have an upper bound. In fact, we can draw another interesting conclusion that the optimum transmit power of IoT devices with RIS is lower than that without RIS, which can illustrate that RIS-aided systems not only improve SE but also save energy. On the other hand, we also conclude that if the phase shifts are not well tuned in the intense LI situation, the random phase shifts method can even be lower by 0.84 bps/Hz than the case without RIS. This underscores the importance of RIS optimization in the urban outdoor environment. Meanwhile, for p=30 dBm, our proposal outweighs the unoptimized RIS approach by 6.23 bps/Hz. It demonstrates that the proposed algorithm still performs well even with an excessively high transmit power.

To further illustrate the merit of the proposal in terms of complexity and power consumption, we list the performance of discrete phase shifts related methods with transmit powers P=30 and p=20 dBm in Table 3.

It can be seen that the EE of the proposal is only 7.4% less than the exhaustive, but the complexity is greatly reduced.

### 4.9. Impact of the Rician Factor

**Figure 12:**
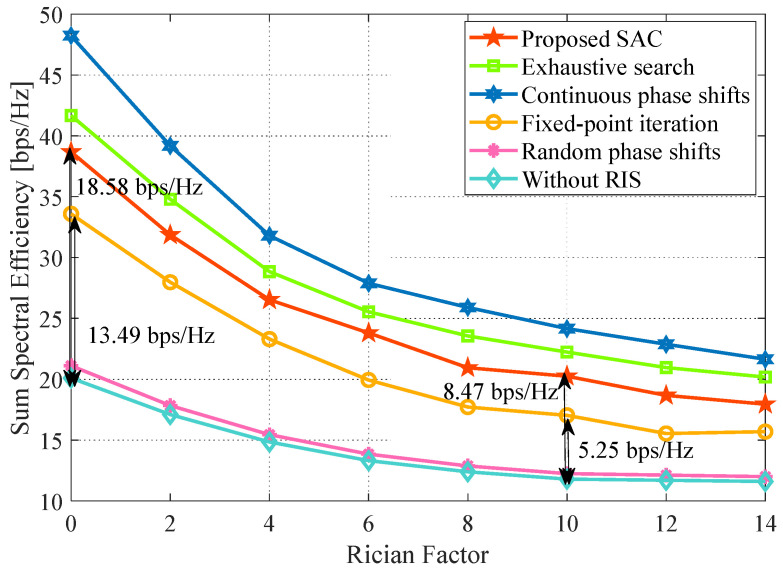
Impact of the rician factor. System parameters: $N_{t}=N_{r}=J=K=3$, b=4, L=16, P=30 dBm, p=20 dBm, x=50 m.

Finally, we evaluate the SE performance under different rician factors in Figure 12. With the increase in the rician factor value, the sum SE decreases, especially for the optimized RIS cases. Moreover, it also can be seen that with ε growth, the gain loss with the proposed algorithm is more evident than the fixed-point iteration method. For instance, the gap between the proposal and the case without RIS is 18.58 bps/Hz at ε=0 and reduces to 8.47 bps/Hz at ε=10, whereas the fixed-point iteration method decreases from 13.49 to 5.25 bps/Hz with the same settings. The reason is that RIS improves performance by reconstructing signals to increase multi-path diversity. A larger ε will not be conducive to promoting multi-path and cause severe co-channel interference due to the enhanced main path. Therefore, the gain brought by multi-path diversity decreases coupled with the increase in ε, and the greater profit deriving from the diversity gain will cause more performance decline under the same level of the enlarged ε. We conclude that the RIS should be deployed in a relatively rich scattering environment to seek optimum performance. Thus, our proposal fairly suits the urban outdoor environment with rich scattering.

## 5. Conclusions

In this paper, considering residual SI, LI, and RIS location, we have proposed a novel DRL-based two-step algorithm practical for two typical urban outdoor scenarios in RIS-aided FD systems. Specifically, we devise a scheme to maximize the sum SE of IoT devices by joint design of the receive, transmitting beamforming matrices and phase shifts vector. Firstly, we decompose the original optimization problem into two subproblems according to the type of optimized variables. We obtain the closed solution of subproblem one by approximating the convex lower bound of the objective function and constraints. Then, we devise a low computational complexity SAC algorithm to solve subproblem two. Simulation results demonstrate that our low-complexity proposal is second only to the upper bound of the discrete phase shifts method and outperforms the fixed-point iteration baseline. Moreover, it is especially advantageous in scenario two with a complicated objective function, proving the superiority of our proposal in the urban outdoor environment.

Since obtaining the perfect CSI of the cascaded channel is idealistic, imperfect CSI should be considered in practice. Moreover, we only indicate that the RIS-aided system is also power-saving from the perspective of SE. We could formulate an EE objective function to investigate further. The above-mentioned is our future work.

## Figures and Tables

**Table 1:** List of abbreviations.

Abbreviation	Definition	Fundamental Usage (If Any)
5G	Fifth generation	
6G	Sixth generation	
AWGN	Additive white gaussian noise	
BS	Base station	
CSI	Channel state information	
DL	Downlink	Downlink communication direction
DRL	Deep reinforcement learning	
EE	Energy efficiency	
FD	Full-duplex	
FDD	Frequency division duplex	
HD	Half-duplex	
IoT	Internet of things	
LI	Loop-interference	Interference caused by the rebounded signal of RIS
LoS	Line-of-sight	
MDP	Markov decision process	
NLoS	None-line-of-sight	
NN	Neural network	
QoS	Quality of service	
RIS	Reconfigurable intelligent surface	
RL	Reinforcement learning	
SAC	Soft actor–critic	
SCA	Successive convex approximation	
SE	Spectral efficiency	
SI	Self-interference	Self-interference of FD BS
SINR	Signal to interference plus noise ratio	
SOC	Second-order cone	
TDD	Time division duplex	
UDI	Uplink to downlink interference	Direct link interference of UL to DL IoT devices
UL	Uplink	Uplink communication direction

**Table 2:** Simulation Hyper-parameters.

Description	Simulation Value
Batch size	256
Replay buffer size	1,000,000
Target update interval	1
Discount rate	0.95
Learning rate for critic network	0.0003
Learning rate for actor network	0.0001
Learning rate for temperature	0.05
Soft update	0.005
Optimizer	Adam
Loss	Mean squared error
Target entropy	−dim(action)
Time steps	40,000

**Table 3:** Performance comparison.

Algorithm	Complexity	EE ((bit/Hz)/Joule)
Proposed SAC	$O({\left(J+K+2\right)}^{2.5}{\left(KN_{t}+JN_{r}+J^{2}+J+K\right)}^{2}+{\left(J+K+2\right)}^{3.5}$ $+2((J+K+L)L_{1}+L_{1}L_{2}+L_{2})+(J+K)L_{1}+L_{1}L_{2}+L_{2}L)$	16.37
Exhaustive search	$O({\left(J+K+2\right)}^{2.5}{\left(KN_{t}+JN_{r}+J^{2}+J+K\right)}^{2}+{\left(J+K+2\right)}^{3.5}+2^{bL})$	17.68
Fixed-point iteration	$O({\left(J+K+2\right)}^{2.5}{\left(KN_{t}+JN_{r}+J^{2}+J+K\right)}^{2}+{\left(J+K+2\right)}^{3.5}$ $+{\left(L+1\right)}^{2}(K^{2}+J^{2}+KJ))$	13.84
Random phase shifts	$O({\left(J+K+2\right)}^{2.5}{\left(KN_{t}+JN_{r}+J^{2}+J+K\right)}^{2}+{\left(J+K+2\right)}^{3.5})$	9.81
Without RIS	$O({\left(J+K+2\right)}^{2.5}{\left(KN_{t}+JN_{r}+J^{2}+J+K\right)}^{2}+{\left(J+K+2\right)}^{3.5})$	9.46

## Data Availability

Data are contained within the article.
